# Exploring the Genomic Architectures of Health, Physical Traits and Antisocial Behavioral Outcomes: A Brief Report

**DOI:** 10.3389/fpsyt.2020.00539

**Published:** 2020-06-25

**Authors:** Jorim J. Tielbeek, Brian B. Boutwell

**Affiliations:** ^1^ Department of Complex Trait Genetics, Center for Neurogenomics and Cognitive Research, Amsterdam Neuroscience, Vrije Universiteit Amsterdam, Amsterdam, Netherlands; ^2^ Department of Criminal Justice and Legal Studies, School of Applied Sciences, University of Mississippi, University, MS, United States; ^3^ John D. Bower School of Population Health, University of Mississippi Medical Center, Jackson, MS, United States

**Keywords:** genetic correlation analysis, genome wide association analysis, antisocial behavior, disease traits, comorbidity

## Abstract

A widely replicated finding across the behavioral sciences is that antisocial behaviors correlate with an array of health problems. Less clear, however, is the precise nature of this association. There is reason to suspect that a direct causal link exists between incarceration—a consequence of some antisocial behaviors—and certain negative health outcomes, for instance. However, it might be the case that broader phenotypes like antisocial behavior may correlate with certain health and physiological traits at a genomic level. We explore this possibility from a theoretical vantage point, while also presenting some preliminary data from existing secondary sources. Tentatively, no significant genetic correlations emerged across a host of health, physiological, and wellbeing outcomes after correction for multiple testing. However, more work is needed exploring this topic. We propose that future studies should make use of larger, more diverse samples and examine the genetic overlap between homogeneous clusters of antisocial behavioral subtypes and disease traits or symptoms.

## Introduction

Prior research has produced well-replicated associations between various health maladies (both psychological and medical) and a range of antisocial behaviors (ASB) (1–4). Overtly aggressive forms of behavior in particular are correlated with an array of diseases; chronic and acute, communicable and non-communicable, and including diabetes, hepatitis, asthma, hyperlipidemia, cancer, and various attendant medical complications such as sepsis (2, 5–7). Children and adolescents with psychiatric diagnoses such as attention deficit/hyperactivity disorder (ADHD)—a key risk factor for crime and ASB—are at an increased risk for adverse health outcomes that can include sleep apnea, diabetes, and obesity (8–10).

While the evidence regarding phenotypic correlation seems clear, less apparent is whether some covariance between ASB and health indicators also exists at a genomic level (11). This becomes possible owing to several reasons, including but not limited to the diffuse heritability of quantitative human traits, as well as arguments made regarding the possibility of “omnigenic” influences in nature (12, 13). The purpose of this brief report, then, is to shed light on possible associations between ASB and a wide swath of relevant health, physiological, and disease traits. The findings to come in this area, regardless of their precise outcome, are important as they will have key implications for research on this topic. To the extent that a genetic correlation exists across traits it both suggests a shared etiology for health and ASB, and also highlights the need to better understand mechanistic pathways from genetic to phenotypic variation (12). Correlated genetic factors must also be controlled in epidemiological and observational studies seeking to explore causal effects running from ASB to health or vice versa (14, 15). An absence of a genetic correlation is also of interest, as it suggests areas where genetic influences are less pleiotropic, and thus will necessarily shape the search for other trait relevant variants for both sets of outcomes independently. Below, we first examine some of the indirect evidence bearing on the current focus. We then transition to the work being done directly on the genomics of antisocial behavior, and discuss reasons why overlap between ASB and health/wellbeing may exist.

### The Correlations Between Health, Disease, and ASB-Relevant Outcomes

There may exist some shared genetic variance between ASB and health/physiological maladies, but empirical evidence on this question is largely lacking or absent entirely. To be sure, there is reason to suspect that such an association *might* exist. Both sets of outcomes—ASB and health—are moderately heritable (13). Given the pleiotropy that exists in the genome, moreover, it seems reasonable to hypothesize that some of the variants associated with health and ASB overlap (12). Finally, phenotypic correlations often reflect genetic correlations to some extent (11). This last point is not fundamental to the hypotheses being tested per se, however, does seem broadly relevant when considering possible overlapping genomic architectures between outcomes correlated at a phenotypic level.

To illustrate our point, consider the limited results gleaned from prior genome wide association (GWA) based studies which relate to the current topic. As we mentioned above, certain cognitive traits/psychological disorders (i.e., ADHD) can function to increase the risk of ASB while also presenting concurrently with certain health maladies (16–18). Hagenaars and colleagues (16) provide a general summary regarding genomic correlations between cognitive traits and health [we direct interested readers specifically to their supplementary tables, and studies such as Brainin et al. (19) and Kovacic et al. (20)]. It should be reiterated, though, that the studies referenced are illustrative, yet only indirectly related to our current focus. Most research to date has not examined the genomic architectures of ASB directly, thus cannot directly elucidate the research question of interest here.

### Better Characterizing the Genomics of Antisocial Behavior

Indeed, to best address the current question a necessary first step involved examining the genomic architectures of various antisocial behaviors. While well-powered genome wide association studies (GWAS) have become widely available in general, comparatively less effort has been focused on antisocial behavioral phenotypes (15, 21). The result, to date, has meant somewhat limited statistical power when examining antisocial behavioral phenotypes (compared to health/disease outcomes). Nonetheless, work in the area is growing, and to address the power problems (as well as other previous limitations), several consortia were formed combining several datasets in order to increase efficiency of research in this area.

Examples of these far-reaching collaborations studying the genetics of ASB include: ACTION (Aggression in Children: Unraveling gene–environment interplay to inform Treatment and InterventION strategies), the Agressotype consortium, BroadABC (Broad Antisocial Behavior Consortium) and EAGLE (Early Genetics and Lifecourse Epidemiology). Using consortia data, we have previously meta-analyzed GWAS data of the non-overlapping cohorts of the BroadABC and EAGLE groups (combined N = 31,968) (22). Despite the identification of a few promising genetic loci (such as chr1:rs2764450; chr2: rs11126630; chr11:rs11215217; and chrX: rs41456347) these large collaborative GWA efforts have not yet produced robust and reproducible genetic variants associated with ASB (22).

Still, these consortia have demonstrated a polygenic contribution of common variants to antisocial behavior and revealed genetic overlap of antisocial behavior with several psychological, psychiatric and addictive outcomes. We demonstrated preliminary evidence of a genetic correlation between reproductively relevant traits and ASB, for instance. In particular, genetic correlation analyses suggested that alleles associated with higher reproductive output were positively correlated with alleles associated with ASB, whereas alleles associated with giving birth later in life were negatively associated (23). Moreover, in a study using a similar research design we found preliminary support for a genetic correlation of ASB with lifetime cannabis use and cigarettes per day, but not with weekly alcohol consumption or ever smoking (24).

## Methods and Results

The current brief report expands on the research just mentioned. To maximize sample size, this study included population-based cohorts with a broad range of antisocial measures, covering both aggressive and non-aggressive domains of antisocial behavior, and with different age groups represented [age ranges for EAGLE included 3 to 15 years old, and BroadABC included ages ranging between 7 and 56 years old; additional details are provided elsewhere, see Pappa et al. (22); Tielbeek et al. (25)]. This brief report represents our first exploration examining the genetic overlap of antisocial behavior, utilizing combined summary data from two consortia [for more detail, see Pappa et al. (22), Tielbeek et al. (25)] with 71 traits derived from the centralized database LD Hub (26) (see Footnote ^1^).

We should momentarily elaborate on the selection and inclusion of health and physiological measures. As mentioned, there are a host of health, physiological, and disease relevant outcomes correlated across numerous prior studies with ASB (see above). As additional GWA summary statistics have become publicly available (see Footnote 1), we opted to cast a wide net when analyzing the possible shared genomic architectures of ASB and health/disease/physiology (as opposed to numerous papers, each smaller in scope).^2^ While desirable in some respects, this approach also further necessitates the need to guard against the possibility of chance findings. With this point in mind, we uncovered no significant (α < 0.0007) genetic correlations after a Bonferonni correction was applied to the data (**Table 1**).

**Table 1:** Genetic correlations (r_g_) between 71 traits (anthropometric, autoimmune, cancer, glycemic, hematological, hormone, kidney, lipids, metal, neurological, sleeping) and broad antisocial behavior.

Trait Category	Phenotype	Ethnicity	r_g_	SE	p	SNP h^2^
Anthropometric	Child birth length	European	0.069	0.212	0.746	0.175
Anthropometric	Child birth weight	European	-0.490	0.251	0.051	0.125
Anthropometric	Body mass index	European	0.201	0.127	0.114	0.191
Anthropometric	Body fat	Mixed	0.291	0.159	0.068	0.109
Anthropometric	Childhood obesity	European	0.252	0.176	0.152	0.400
Anthropometric	Extreme bmi	European	0.165	0.144	0.252	0.694
Anthropometric	Extreme height	European	0.062	0.110	0.575	0.123
Anthropometric	Extreme waist-to-hip ratio	European	0.109	0.222	0.623	0.353
Anthropometric	Height_2010	European	0.047	0.090	0.599	0.285
Anthropometric	Obesity class 1	European	0.185	0.121	0.127	0.217
Anthropometric	Obesity class 2	European	0.210	0.131	0.108	0.184
Anthropometric	Obesity class 3	European	0.216	0.172	0.209	0.122
Anthropometric	Overweight	European	0.136	0.118	0.250	0.110
Anthropometric	Hip circumference	European	0.022	0.091	0.812	0.129
Anthropometric	Infant head circumference	European	0.125	0.254	0.622	0.228
Anthropometric	Waist circumference	European	0.114	0.095	0.233	0.122
Anthropometric	Waist-to-hip ratio	European	0.303	0.126	0.016*	0.114
Anthropometric	Difference in height between adolescence and adulthood age 14	European	-0.069	0.272	0.800	0.474
Anthropometric	Difference in height between childhood and adulthood age 8	European	-0.120	0.217	0.581	0.320
Anthropometric	Sitting height ratio	European	0.320	0.184	0.082	0.227
Anthropometric	Height Females at age 10 and males at age 12	European	-0.026	0.179	0.886	0.431
Anthropometric	Birth weight	European	-0.136	0.121	0.260	0.098
Autoimmune	Eczema	Mixed	0.400	0.223	0.072	0.069
Autoimmune	Crohns disease	European	-0.003	0.141	0.983	0.478
Autoimmune	Inflammatory Bowel Disease (Euro)	European	-0.228	0.152	0.133	0.300
Autoimmune	Ulcerative colitis	European	-0.391	0.189	0.038*	0.224
Autoimmune	Asthma	European	0.051	0.186	0.784	0.142
Autoimmune	Rheumatoid Arthritis	European	0.223	0.144	0.122	0.157
Autoimmune	Multiple sclerosis	European	0.143	0.279	0.609	0.073
Autoimmune	Systemic lupus erythematosus	European	0.089	0.185	0.629	0.408
Autoimmune	Primary biliary cirrhosis	European	0.132	0.221	0.549	0.365
Autoimmune	Celiac disease	European	0.033	0.265	0.902	0.308
Autoimmune	Primary sclerosing cholangitis	Mixed	-0.556	0.256	0.030*	0.399
Cancer	Lung cancer (all)	European	0.088	0.185	0.636	0.130
Cancer	Lung cancer (squamous cell)	European	0.228	0.310	0.463	0.048
Cancer	Lung adenocarcinoma	European	0.124	0.309	0.688	0.025
Cancer	Squamous cell lung cancer	European	0.153	0.263	0.560	0.037
Cancer	Lung cancer	European	0.061	0.173	0.727	0.323
Glycemic	Type 2 Diabetes	European	-0.143	0.162	0.378	0.090
Glycemic	Fasting glucose main effect	European	0.052	0.150	0.729	0.104
Glycemic	Fasting insulin main effect	European	0.276	0.217	0.202	0.070
Glycemic	Fasting proinsulin	European	-0.274	0.287	0.340	0.189
Glycemic	2hr glucose adjusted for BMI	European	0.033	0.267	0.901	0.109
Glycemic	HbA1C	European	0.264	0.257	0.305	0.064
Glycemic	HOMA-B	European	0.346	0.213	0.104	0.087
Glycemic	HOMA-IR	European	0.574	0.270	0.034*	0.068
Haemotological	Heart rate	Mixed	0.116	0.143	0.416	0.082
Haemotological	Mean platelet volume	European	0.155	0.182	0.396	0.323
Haemotological	Platelet count	European	-0.130	0.152	0.391	0.132
Hormone	Leptin_adjBMI	European	-0.101	0.214	0.637	0.094
Hormone	Leptin_not_adjBMI	European	0.054	0.218	0.803	0.098
Kidney	Urinary albumin-to-creatinine ratio (non-diabetes)	European	-0.191	0.240	0.426	0.055
Kidney	Chronic Kidney Disease	Mixed	-0.152	0.230	0.508	0.020
Kidney	Serum creatinine (non-diabetes)	Mixed	-0.177	0.123	0.150	0.115
Kidney	Serum creatinine	Mixed	-0.144	0.117	0.219	0.108
Kidney	Serum cystatin c	Mixed	-0.207	0.165	0.210	0.170
Kidney	Urinary albumin-to-creatinine ratio	European	-0.152	0.222	0.495	0.048
Lipids	HDL cholesterol	European	0.009	0.121	0.939	0.120
Lipids	LDL cholesterol	European	0.169	0.130	0.196	0.105
Lipids	Triglycerides	European	0.129	0.114	0.257	0.174
Lipids	Total Cholesterol	European	0.229	0.127	0.070	0.135
Metal	Transferrin	European	0.190	0.224	0.396	0.163
Metal	Ferritin	European	0.173	0.307	0.573	0.094
Neurological	Alzheimers disease	European	0.044	0.211	0.834	0.051
Neurological	Parkinsons disease	European	0.072	0.146	0.622	0.419
Neurological	Amyotrophic lateral sclerosis	European	0.190	0.338	0.575	0.033
Sleeping	Sleep duration	European	-0.073	0.152	0.630	0.056
Sleeping	Chronotype	European	0.000	0.119	0.998	0.101
Sleeping	Insomnia^a^	European	0.425	0.191	0.026*	0.047
Sleeping	Insomnia^b^	European	0.300	0.157	0.057	0.133
Sleeping	Excessive daytime sleepiness	European	-0.289	0.174	0.097	0.052

SNP (single nucleotide polymorphism); h^2^: narrow-sense heritability based on all SNPs; r_g_: genetic correlation with ASB. Asterisks (*) indicate a nominal significant genetic correlation (p<.05). for a, see (27); for b, see (28).

Consideration of findings reaching a level of nominally significant (α < 0.05) results revealed that—with increasing sample size—anthropometric risk factors such as high waist-to-hip ratio (r_g_ = 0.30, P = 0.016) may correlate with genetic risk of ASB. Moreover, the potential positive genetic overlap of ASB with insomnia (r_g_ = 0.43, P = 0.026) and HOMA-IR (Homeostatic Model Assessment for Insulin Resistance, r_g_ = 0.57, P = 0.034) is noteworthy [for a related reference, see also Rolling et al. (6)], as well as the negative genetic correlation with autoimmune diseases ulcerative colitis (r_g_ = −0.39, P = 0.038) and primary sclerosing cholangitis (r_g_ = −0.56, P = 0.030). It is key to emphasize, however, that although we report the above moderate to strong genetic correlations, they may in fact reflect *false positive* findings—as they did *not* survive correction for multiple testing. The large standard errors, it is worth noting, reflect the relatively low GWAS sample size for ASB, rendering these results in need of further replicative efforts with larger sample sizes. Finally, in an effort to be as exhaustive as possible beyond our initial analyses, and by making use of the same LD Hub web interface, we calculated the genetic correlations of ASB with an additional 597 health and physiologically relevant traits of the UK Biobank which also recently became available (**Supplementary Table S1**; see also our footnote above concerning the inclusion of these additional traits). Similar to our primary findings, none of these correlations survived correction for multiple testing (α < 8.4 × 10^−5^).

## Discussion

What this preliminary exploration suggests is that ASB and various health outcomes share virtually no genomic overlap, beyond what might be considered at this point as “nominally significant.” Moreover, these findings further suggest that phenotypic associations between ASB and health/physiological outcomes are perhaps phenotypically causal, or mutually impacted by some third *environmental* variable (i.e., spurious, but owing to environmental variables which might include a range of factors such as socioeconomic status, educational attainment, etc.). Keeping that mind, we have several recommendations for future research endeavors examining the overlap of ASB and health/physiological adversities.

### Enlarging and Diversifying Samples

With growing sample sizes, future polygenic risk scores (PRS) will improve in their prediction of antisocial behavioral traits on the basis of genetic data. Moreover, future PRS for disease outcomes may still be capable of elucidating the risk of ASB and vice versa [see Demontis et al. (9)]. Well-powered PRS, such as for Type 1 Diabetes, have great clinical and research potential and have been found useful for classifying adult incident diabetes type and improving newborn screening (29). Despite the great potential of PRS and precision medicine, a major limitation is the Eurocentric bias and the lack of diversity in genetic studies. Indeed, the underrepresentation of non-European populations in GWAS is problematic as it limits the generalizability and predictive value of PRS for these populations (30).

### Examining Homogeneous Clusters

Genetic correlations are computed on the basis of GWA summary statistics of the traits of interest. GWA research designs in psychopathology often employ sum-scores or case-control status summarizing a list of items or symptoms. Items included in these types of composite scores can vary considerably, with some examples including: ‘hit others when provoked’, ‘blamed others’ and ‘had trouble keeping a job’, yet all of them load on the broader construct of antisocial behavior (22). Prior work on neuroticism, however, suggested that the items included were genetically heterogeneous, and thus argued that future studies should examine the trait from the standpoint of homogeneous clusters of relevant alleles (31). To that end, future scholars may find the framework of genomic SEM (structural equation modeling) useful in delineating homogenous clusters (32). Taken together, then, the above recommendations will likely be worthwhile, as it will be important to examine genetic overlap between GWAS summary stats of *subtypes* of ASB (or even on the item level) with disease traits or symptoms in future research.

### Exploring Causality

With the help of well-powered GWAS it has become increasingly clear that most genes influence multiple biological pathways and traits—a phenomenon commonly termed pleiotropy (12). Owing to this issue, it becomes crucial to overcome unmeasured confounding and distinguish between causal and spurious risk factors (33, 34). Despite many epidemiological studies being non-experimental, a research technique called Mendelian randomization (MR)—which is a version of the commonly utilized design known as instrumental variables—can strengthen causal inference by considering genes as the instruments in the design (34). Stated differently, MR studies operate by making use of genetic variants as proxies for the actual (environmental) risk factor of interest (34). The number of studies utilizing this approach is expanding rapidly, but as a note of caution (echoed previously in prior work) future MR studies on this topic should carefully assess the plausibility of the underlying assumptions when implementing the technique, and preferably combine various MR methods whenever possible (33).

### Mechanisms and Gene–Environment Interplay

For the traits that might evince *preliminary* evidence of a genetic correlation (if the nominal levels of significance are meaningful), much more work—with more statistical power—is needed to understand the mechanisms of such associations. We hesitate to speculate widely, but one possibility could be that the genetic influences shared across traits operate widely on immune system functioning as well as general functioning in the central nervous system. The downstream effect then would be the emergence of both psychological and physiological conditions at certain points in the life course (16). Additionally, one cannot discount the relevance of gene–environment interplay in two forms: gene–environment interaction (G × E) and gene–environment correlation (rGE) [in general, see Leppert et al. (35); Ruisch et al. (36); Ruisch et al. (37)].

Indeed, one can envision scenarios such that the impact of trait relevant genes is calibrated based on exposure to environmental insults, including disease or infectious agents [i.e., G × E; for indirectly related examples see Ruisch et al. (36); Ruisch et al. (37)]. Moreover, traits which vary in part because of genetic variation—in this case ASB—might perhaps operate to increase risk of exposure to infectious agents or more broadly, situations where health outcomes could be compromised (36, 37). Future research on this topic will doubtless benefit from exploring the relevancy of rGEs for the intersection of health and ASB. Finally, we should note that interaction with other alleles at additional loci, too, will likely be important for understanding the complex underpinnings of these phenotypes and why their genomic architectures could potentially overlap. In short, the number of research hypotheses awaiting investigation in this area is long, detailed, and it would seem, quite timely.

## Data Availability Statement

The raw data supporting the conclusions of this article will be made available by the authors, without undue reservation, to any qualified researcher.

## Author Contributions

BB was responsible for the conception of the work and drafted the first version. JT performed the statistical analyses and revised it critically for important intellectual contents. All authors contributed to the article and approved the submitted version. 

## Conflict of Interest

The authors declare that the research was conducted in the absence of any commercial or financial relationships that could be construed as a potential conflict of interest.

## Footnotes

1 With additional health traits—which we became aware of after our initial round of peer review—included in the supplementary tables. We opted to expand our focus to include these traits as a result of both reviewer feedback, and the relevancy of the traits for the current research question.

2 We wish to thank an anonymous reviewer for prompting us to consider expanding our focus, given the wider associations between antisocial behavioral outcomes and health/physiologically relevant variables.
